# Intranasal Vaccination with Recombinant TLR2-Active Outer Membrane Vesicles Containing Sequential M2e Epitopes Protects against Lethal Influenza a Challenge

**DOI:** 10.3390/vaccines12070724

**Published:** 2024-06-29

**Authors:** Nisha Kannan, Annette Choi, Mariela A. Rivera De Jesus, Peter Male Wei, Julie Marie Sahler, Stephanie Marie Curley, Avery August, Matthew P. DeLisa, Gary R. Whittaker, David Putnam

**Affiliations:** 1 Meinig School of Biomedical Engineering, Cornell University, Ithaca, NY 14853, USA; nk489@cornell.edu (N.K.); mar524@cornell.edu (M.A.R.D.J.); twm86@cornell.edu (P.M.W.); smc344@cornell.edu (S.M.C.); 2 Department of Microbiology and Immunology, Cornell University, Ithaca, NY 14853, USA; ac2868@cornell.edu (A.C.); js2628@cornell.edu (J.M.S.); averyaugust@cornell.edu (A.A.); gary.whittaker@cornell.edu (G.R.W.); 3 Smith School of Chemical and Biomolecular Engineering, Cornell University, Ithaca, NY 14853, USA; matthew.delisa@cornell.edu

**Keywords:** intranasal vaccine, influenza, OMV vaccine

## Abstract

Influenza is a highly contagious respiratory disease, resulting in an estimated 3 to 5 million cases of severe illness annually. While most influenza vaccines are administered parenterally via injection, one shortcoming is that they do not generate a strong immune response at the site of infection, which can become important in a pandemic. Intranasal vaccines can generate both local and systemic protective immune responses, can reduce costs, and enhance ease of administration. Previous studies showed that parenterally administered outer membrane vesicles (OMVs) that carry sequences of the M2e protein (OMV-M2e) protect against influenza A/PR8 challenge in mice and ferrets. In the current study, we measured the effectiveness of the intranasal route of the OMV-M2e vaccine against the influenza A/PR8 strain in mice. We observed high anti-M2e IgG and IgA titers post-challenge in mice vaccinated intranasally with OMV-M2e. In addition, we observed a Th1/Tc1 bias in the vaccinated mice, and an increased Th17/Tc17 response, both of which correlated with survival to A/PR8 challenge and significantly lower lung viral titers. We conclude that the intranasal-route administration of the OMV-M2e vaccine is a promising approach toward generating protection against influenza A as it leads to an increased proinflammatory immune response correlating with survival to viral challenge.

## 1. Introduction

Protection against seasonal influenza remains a challenge, with annual deaths globally ranging between 294,000 and 518,000 [1]. The infection is particularly fatal to infants, the elderly, pregnant women, and patients with chronic diseases [2]. Current vaccines against influenza target the immunodominant but highly variable glyco-proteins hemagglutinin (HA) and neuraminidase (NA), requiring an annual cocktail of strain-specific vaccines [3]. A strain-agnostic vaccine, colloquially termed a “universal vaccine”, is desired in order to obviate the need for seasonal vaccines, to reduce vaccine/strain subtype mismatch, and to improve vaccination coverage [4]. Approaches to achieving universality are categorized either as the induction of cross-reactive neutralizing antibodies or a strong cellular response against the more conserved and concealed interior viral proteins [5]. Regardless of its design, the goal of a universal vaccine against influenza is to induce protective immunity against all influenza virus subtypes [5].

The extracellular domain of Matrix 2 protein (M2e) is an evolutionarily conserved region in influenza A viruses, and it is a promising antigen in a universal influenza vaccine [6]. Infected epithelial cells present and expose M2e to the immune system [7] although influenza infection generally generates low anti-M2e antibody titers compared to those generated against HA and NA antibodies [8]. In addition, unlike neutralizing anti-HA and anti-NA antibodies, anti-M2e antibodies clear infection through antibody-dependent cell-mediated cytotoxicity (ADCC) and phagocytosis (ADCP) [9,10]. The mechanisms of ADCC and ADCP involve natural killer cells and macrophages, which recognize the Fc regions of the antigen-bound antibody and eliminate infected cells through either cell-mediated cytotoxicity or phagocytosis [10,11]. Because the M2e sequence is relatively conserved compared to HA and NA sequences, and because anti-M2e antibodies can confer protection, a number of universal influenza vaccine candidate designs include the M2e sequence [12,13].

Bacterial outer membrane vesicles (OMVs) are nanoscale structures that constitutively bud from bacteria, particularly Gram (-) bacteria. Because they are bacterially derived, OMVs can be recombinantly engineered to carry pathogen-specific antigens and to carry pathogen-associated molecular patterns to impart engagement with the innate immune system [14,15]. OMVs that contain pathogen-specific antigens are highly immunogenic but non-infectious, making them a promising platform for effective vaccines [15]. However, lipopolysaccharide, which provides the structural vesicle architecture of natural OMVs, is a TLR4 agonist, which precludes the parenteral administration of these OMVs without severe adverse effects. ClearColi (CC), an *E. coli* strain derived from BL21, is engineered to produce a lipopolysaccharide variant that does not robustly trigger TLR4, making it amenable to parenteral administration and a potentially viable vaccine delivery vehicle [16]. In work previously published from our lab, CC-derived OMVs carrying an M2e antigen (CC-OMV-M2e) protected against influenza challenge when administered via subcutaneous injection in both a mouse and a ferret infection model [17,18].

Most vaccines are administered via injection (i.e., intramuscular, intradermal, or subcutaneous) to directly access antigen-presenting cells (APCs) (i.e., dendritic cells) that reside in large numbers under the skin. Dendritic cells serve a protective function by capturing antigens, which induces their maturation and subsequent migration to the draining lymph nodes [19]. Intranasally delivered vaccines are a more patient-preferred route of administration and induce both a local and a systemic response [19,20,21]. Here, we show that the intranasal route of administration is a potentially viable avenue for vaccination using OMVs as an antigen carrier [15,22,23]. We establish that a CC-OMV-M2e vaccine delivered intranasally in mice induces protection against influenza infection. The intranasally administered vaccine elicited significant anti-M2e IgG and IgA titers in CC-OMV-M2e-vaccinated mice compared to controls (PBS, CC-OMV). In addition, we observed vaccine-induced increases in the frequency of Th1 and Th17 cells that may be involved in the protection of CC-OMV-M2e-vaccinated mice against influenza infection.

## 2. Materials and Methods

### 2.1. OMV Production and Characterization

OMVs were prepared and isolated as described previously [24]. M2e4xHet comprises four M2e variants separated by glycine-serine linker [18]. Briefly, hypervesiculating ClearColi (CC) was transformed with a pBAD plasmid expressing ClyA-M2e4xHET (CC-OMV-M2e). Overnight cultures of single colonies from each of the transformed strains were sub-cultured into 4 × 75 mL of Luria–Bertani (LB) medium (Thermo Fisher Scientific, Waltham, MA, USA) supplemented with 25 μg/mL chloramphenicol (Thermo Fisher Scientific) and 50 mg/mL kanamycin (Thermo Fischer Scientific). The cultures were grown to mid-log phase (OD_600_ ~ 0.5–0.6), at which time protein expression was induced with 0.2% L-arabinose (Sigma-Aldrich, St. Louis, MO, USA). Cell-free culture supernatants were collected 16–20 h post-induction and pelleted at 4000 rpm and the supernatants were filtered through a 0.2 μm filter. OMVs were isolated through ultracentrifugation (Beckman-Coulter, Brea, CA, USA; TiSW28 rotor; 26,000 rpm; 3 h; 4 °C) and resuspended in PBS. Dynamic light scattering measurements were made using a Malvern Zetasizer Nano ZS, Worcestershire, UK (refractive index: 1.367, dispersant: PBS, concentration: 0.4 mg/mL, and temperature: 25 °C) (Figure S1B). The protein content of the OMV preparations was determined using a BCA assay (Thermo Fisher Pierce), and antigen content was determined via semi-quantitative Western blot (WB). To detect ClyA-M2e4xHet, both anti-His (clone HIS-1) antibody (Sigma-Aldrich) and anti-ClyA antibody (Thermo Fisher) were used. WBs were developed using chemiluminescence and imaged with ChemiDoc Touch Imaging System (Bio-Rad) (Figure S1A).

### 2.2. Immunization and Infection

Female BALB/c mice (5–7 weeks old) were purchased from the Jackson Laboratory and housed in pathogen-free conditions in the East Campus Research Facility Animal Facility at Cornell University. All animal experiments were approved by the Institutional Animal Care and Use Committee at Cornell University, Ithaca, NY, USA and methods were performed in accordance with the relevant guidelines and regulations.

Mice were immunized intranasally with 50 µL of vaccine under isoflurane anesthesia. Treatment groups (PBS (*n* = 5), CC-OMV (*n* = 5), CC-OMV-M2e (*n* = 5)) received 40 μg of total OMV protein, which contained 14.5 µg of the M2e antigen (amounting to 36.4% of total protein) in PBS. Mice administered PBS intranasally were used as negative controls (*n* = 5). Two doses for all groups (prime and boost) were administered 4 weeks apart. All mice were weighed and observed following the prime and boost dose.

Influenza A/Puerto Rico/8/1934 (PR8) virus (BEI Resources, Manassas, VA, USA) titrated in PBS was used to challenge each group of mice 8 weeks following prime vaccination. The PR8 virus was titrated from an original stock of 1.3 × 10^6^ PFU/mL. The inoculated titer was determined using a plaque assay. Briefly, 3.5 × 10^5^ cells/mL MDCK cells, grown in DMEM medium (Thermo Fisher), were seeded in a 12-well plate (Corning, Corning, NY, USA). Serially diluted virus was added to the cells and incubated at 37 °C for 1 h. Agar overlay (0.3% Oxid agar in DMEM with 20 mg/mL trypsin) was added and incubated at 37 °C until plaque was visible. Cells were fixed with 4% PFA and stained with crystal violet, and plaques were visually counted.

Mice were administered 50 PFU/50 µL via intranasal injection under isoflurane anesthesia. Mice were weighed daily following influenza infection. Any mouse with more than 30% weight loss, or exhibiting signs of severe distress, was euthanized.

### 2.3. Quantification of Lung Viral Titers

To compare viral titers of influenza-challenged mice, post-euthanasia, lungs were collected and immediately immersed in Trizol LS (Thermo Fisher) and stored at −80 °C. Total RNA was extracted, and real-time quantitative PCR (rt-qPCR) was performed with iTaq universal probes one-step kit (BioRad). Influenza A cross-reacting primers designed based on WSN Nucleoprotein (NP) were used (forward: CCAAATGAGAATCCAGCACAC; reverse: CCACTTTCGTCCCTCTGATG). The Ct value of the target samples was converted to the viral copy number using the standard curve and normalized to the RNA concentration, which was determined using a Qubit 4 fluorometer (Thermo Fisher).

### 2.4. Collection of Mouse Sera and Detection Serum Antibodies

Serum was collected one day before the prime and booster doses, 2 weeks after the booster dose, and post-termination for all groups. ELISAs were performed as previously described [17]. Briefly, polystyrene microtiter 96-well Nunc Maxisorp plates (Nalgene Nunc) were coated (2 µg/mL M2e peptide (Sequence: SLLTEVETPIRNEWGCRCNDSSD), 12 h, 37 °C), blocked (5% milk in PBS, 1 h, 25 °C), and then incubated with 8-fold serial dilutions of serum in triplicate (2 h, 37 °C). Plates were subsequently incubated with biotin-conjugated antibody (1:1000 dilution; IgG, IgG1, IgG2a or IgA) (eBioscience, San Diego, CA, USA) (1 h, 37 °C), incubated with HRP-avidin (1:1000 dilution; 0.5 h, 37 °C), and developed in the dark for 20 min with TMB (Thermo Fisher Scientific); the reaction was stopped with 25 µL 2N H_2_SO_4_, and absorbance was read at 450 nm and 570 nm. The plates were washed at least three times with wash buffer (0.05% Tween 20 in PBS) between each step. Antibody titers were determined, each as the highest dilution in which the sample gave a consistent signal greater than the average plus three times the standard deviation of the background at the same dilution.

### 2.5. Splenocyte Isolation and Culture

Mice were euthanized and spleens were harvested, then mechanically homogenized using the back of a 5 mL syringe and filtered through a 100 µm strainer (Corning). Red blood cells were lysed with 1X red blood cell lysis buffer (eBioscience) for 5 min, then each sample was centrifuged and the supernatant was removed. Cells in the resulting pellet were resuspended in RPMI 1640 media (Thermo Fisher Scientific) supplemented with 2 mM L-glutamine, 10% FBS (Gibco), 100 U/mL penicillin, 100 µg/mL streptomycin (Gibco, Waltham, MA, USA), 5 mM HEPES (Sigma-Aldrich), and 50 µM beta-mercaptoethanol (Thermo Fisher Scientific). Cells were maintained in complete RPMI media at 37 °C and 5% CO_2_ for the remainder of the experiment. Cells were seeded in 96-well plates (VWR international, Radnor, PA, USA) (three technical replicates per spleen) at a density of 2.5 × 10^5^ cells per well for the flow cytometry and Luminex analysis. Cells were seeded and re-stimulated ex vivo with CC-OMV-M2e (1 µg/mL) or media as an unstimulated control.

### 2.6. Flow Cytometric Analysis of Effector T Cell Cytokine

After 31 h of splenocyte re-stimulation, 1X protein transport inhibitor cocktail (eBioscience) was added and the cells were further incubated for 5 h at 37 °C. After 5 h, cells were transferred to V-bottom 96-well plates (Corning), centrifuged, and had the supernatants removed. Live/Dead viability dye was added (e780, eBioscience) and incubated for 30 min on ice. Cells were washed with flow wash buffer (2% FBS in PBS), then stained with surface marker antibodies FITC-anti-mouse CD3 (Biolegend, San Diego, CA, USA), Brilliant Violet anti-mouse CD4 (Biolegend), and eFluor 450 CD8a (eBioscience) for 20 min on ice. Cells were washed, then fixed in 2% paraformaldehyde (Thermo Fisher Scientific) in PBS for 20 min. After fixation, cells were washed with flow wash buffer, then incubated for 10 min in permeabilization buffer (1% FBS and 0.05% saponin in PBS) for intracellular staining. Cells were stained for 20 min with Phycoerythrin (PE) anti-mouse IFN-γ antibody (Biolegend), PerCP-Cy 5.5 anti-mouse TNF-α antibody (Biolegend), and PE/Cy7 anti-mouse IL-17A antibody (Biolegend). Cells were again washed, then resuspended in flow wash buffer before analysis through flow cytometry. Sample data were acquired using an Attune NxT (Invitrogen, Waltham, MA, USA) and analyzed with FlowJo Software (v10.8.1).

### 2.7. Total Cytokine Secretion Analysis Using Luminex

After 36 h of splenocyte re-stimulation, the 96-well plates were centrifuged at 1500 rpm for 5 min, and culture supernatant was harvested and stored at −80 °C. Cell culture supernatant (25 μL) was analyzed in an Invitrogen ProcartaPlex Panel Kit (Invitrogen) (17-Plex Mouse Cytokine/Chemokine Panel). The cytokines studied included GM-CSF, IFN-γ, IL-1 β, IL-2, IL-4, IL-5, IL-6, IL-12p70, IL-13, IL-18, TNFα, IL-9, IL-10, IL-17A (CTLA-8), IL-22, IL-23, and IL-27. The concentration of each analyte was calculated using the Bio-Plex manager 6.0 software with a 5-parameter curve-fitting algorithm applied for standard curve.

### 2.8. Statistical Analysis

Post-vaccination serum data are represented as log-transformed antibody titers. Error bars represent 95% CI of geometric means with four to five mice in each group. Data were further compared using two-way ANOVA and Tukey’s post hoc test. Post-termination serum data are represented as log-transformed antibody titers. Error bars represent 95% CI of geometric means with four to five mice in each group. Data were further compared using two-way ANOVA and Sidak post hoc test.

For flow cytometry and Luminex data, the results are presented as means + SEM with four to five mice in each group. The values were analyzed through ordinary one-way ANOVA followed by a Tukey or Sidak post hoc test. All statistical analyses were conducted using Prism8 software (GraphPad Software ver 10).

## 3. Results

### 3.1. Intranasal Vaccination with CC-OMV-M2e Protects against Lethal Influenza A/PR8 Challenge in BALB/c Mice

**Figure 1:**
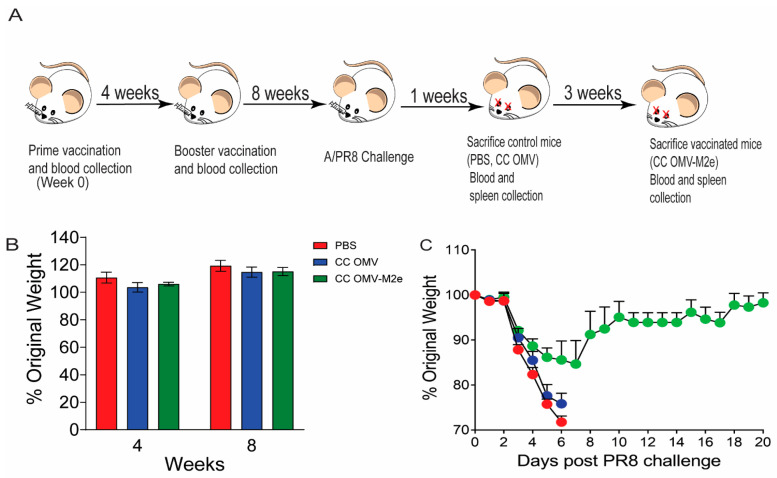
Immunization schematic and prime and booster body weight: (**A**) Balb/c mice were vaccinated through the intranasal route at week 0 and a booster dose was administered at week 4. At week 8, mice were intranasally challenged with the A/PR8 strain of influenza. Blood was collected at weeks 0, 4, 6, and 8. One week post-challenge, control mice (PBS, CC-OMV) were sacrificed and blood and spleen were harvested. Three weeks post-challenge, vaccinated mice (CC-OMV-M2e) were sacrificed, and blood and spleen were harvested. (**B**) Mice were weighed post-prime and boost immunization. Error bars represent means ± SEMs. (**C**) Weight loss in mice challenged with a lethal dose of 50 PFU influenza A/PR8 (PBS-vaccinated *n* = 5, CC-OMV-vaccinated *n* = 5, CC-OMV-M2e-vaccinated *n* = 5) was recorded daily. Any mouse with more than 30% weight loss, or exhibiting signs of severe distress, was euthanized. Error bars on mortality curves represent means ± SEMs.

To evaluate the efficacy of the intranasal vaccine route of administration in Balb/c mice, three groups of mice were vaccinated with PBS (sham control), CC-OMV (negative control), and CC-OMV-M2e (Figure 1A). The OMV constructs were engineered from the CC *E.coli* strain, which produces modified lipopolysaccharide, making it free of significant endotoxin effects [16]. A previous study from our group showed that an OMV-based vaccine containing the M2e antigen generated using the CC strain and administered via the subcutaneous route stimulated a protective immune response in mice against influenza and retained TLR2 agonist activity [17]. Building on this earlier work, we considered the possibility that intranasal prime and boost immunization could be similarly effective. Immunized mice were weighed to determine whether the low degree of pyrogenicity in the CC-OMV-M2e formulation could lead to weight loss. CC-OMV-M2e-vaccinated mice maintained normal activity and appearance and experienced no weight loss after either dose and were comparable to mice receiving intranasally administered sham PBS and CC-OMV formulations (Figure 1B).

**Figure 2:**
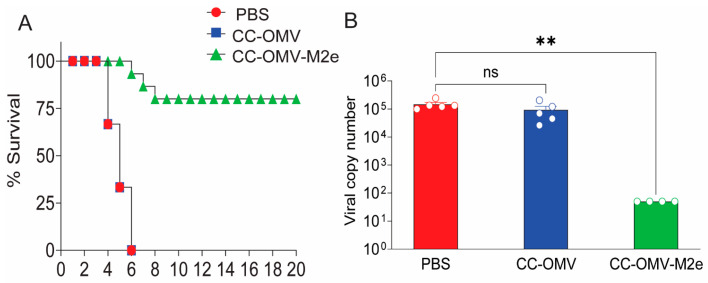
Survival curve and lung viral load. (**A**) Survival of mice challenged with a lethal dose of 50 PFU influenza A/PR8 (PBS-vaccinated *n* = 5, CC-OMV-vaccinated *n* = 5, CC-OMV-M2e-vaccinated *n* = 5). Kaplan–Meier survival curves were analyzed with a log-rank test using the Mantel–Cox method (*p* < 0.0001). Error bars on survival curves represent means ± SEMs. (**B**) PR8 viral copy number from lungs excised on day 6 post-challenge for the controls (PBS and CC-OMV only) and CC-OMV-M2e (day 20 post-challenge) were quantified via RT-PCR. Error bars on the viral copy number represent means ± SEMs. ** *p* < 0.01, ns-non significant, as per ordinary one-way ANOVA and Tukey’s post hoc test.

Eight weeks post-prime immunization, all groups were challenged with a lethal dose (50 PFU) of influenza A strain PR8. Intranasal immunization with CC-OMV-M2e protected 80% of mice vaccinated as one mouse in the CC-OMV-M2e group required euthanasia due to weight loss exceeding 30% original body weight (Figure 1C and Figure 2A). Though the CC-OMV-M2e mice showed weight loss, this result was expected as M2e-based vaccines are infection-permissive, requiring the Fc-dependent clearance of infected cells for protection [11]. Additionally, the average lung viral copy number of CC-OMV-M2e-vaccinated mice was approximately 2000-fold lower than that of the controls (PBS, CC-OMV) post-challenge (Figure 2B). Taken together, the results confirm that the intranasal route is a promising approach for protection against a lethal influenza challenge.

### 3.2. Intranasal Vaccination with CC-OMV-M2e Elicits anti-M2e IgG and IgA Titers Post-Infection and Protects Balb/C Mice from Lethal Influenza A/PR8 Challenge

**Figure 3:**
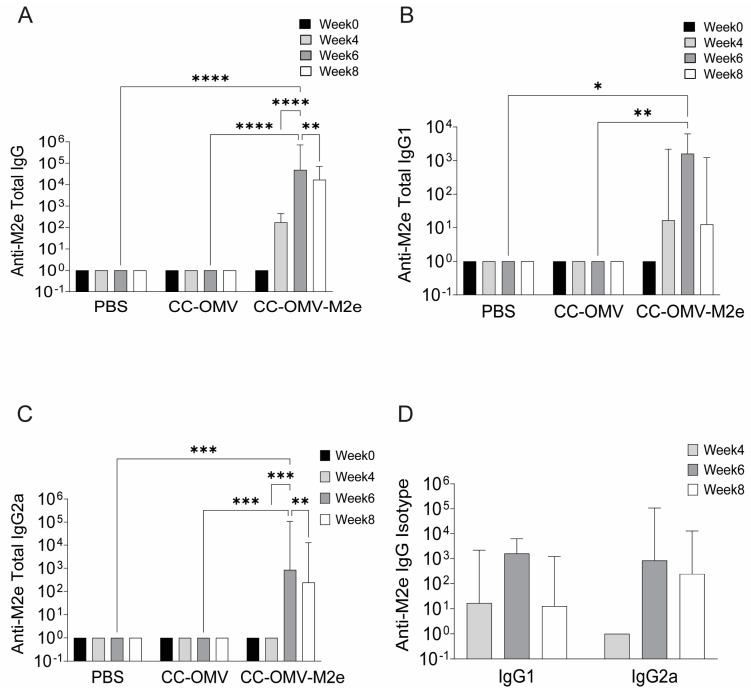
Anti-M2e antibodies post-vaccination: serum was collected from mice at week 0, week 4, week 6, and week 8 and tested for (**A**) total IgG, (**B**) IgG1, and (**C**) IgG2a antibodies against 2 µg/mL M2e peptide using ELISA. Data are represented as log-transformed antibody titers. Error bars represent 95% CI of geometric means with five mice in each group (CC-OMV-M2e *n* = 5, CC-OMV *n* = 5, PBS *n* = 5). * *p* < 0.05, ** *p* < 0.01, *** *p* < 0.001, and **** *p* < 0.0001 as per two-way ANOVA and Tukey’s post hoc test. (**D**) Anti-M2e IgG1 and IgG2a titers from mice 4, 6, and 8 weeks post-prime CC-OMV-M2e immunization. Data are represented as log-transformed antibody titers; error bars indicate 95% CI of geometric means (PBS-vaccinated *n* = 5, CC-OMV-vaccinated *n* = 5, CC-OMV-M2e-vaccinated *n* = 5).

**Figure 4:**
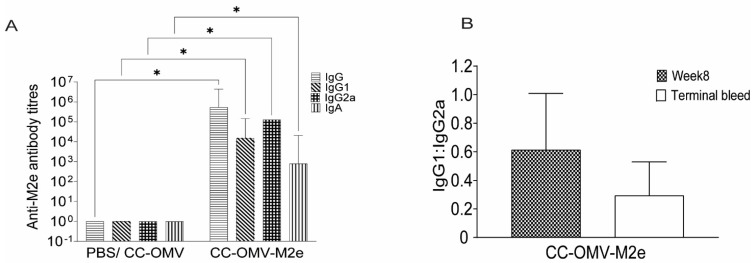
Anti-M2e antibodies post-challenge: (**A**) serum was collected on termination and tested for total IgG, IgG1, IgG2a, and IgA antibodies against M2e peptide using ELISA. Data are represented as log-transformed antibody titers. Error bars represent 95% CI of geometric means with four to five mice in each group (CC-OMV-M2e *n* = 4, CC-OMV *n* = 5, PBS *n* = 5). * *p* < 0.05 as per two-way ANOVA and Sidak post hoc test. (**B**) Serum from CC-OMV-M2e-vaccinated mice was assessed at week 8 and terminal bleeds to compare the IgG1:IgG2a ratios. Data are represented as ratios ±SEMs.

The immune response to mucosal immunization with CC-OMV-M2e through intranasal administration was quantified through antibody response pre- and post-influenza challenge. Serum samples from all groups at weeks 0, 4, 6, and 8 were assessed. Mice vaccinated with CC-OMV-M2e all developed high and statistically significant titers of anti-M2e IgG at weeks 4, 6, and 8 post-initial vaccination compared to the controls (PBS, CC-OMV) (Figure 3A). Anti-M2e IgG isotypes were also compared between weeks 4, 6, and 8 and increased titers were observed (Figure 3B–D). Post-influenza-challenge antibody titers were measured in both vaccinated and control mice. Significantly higher total anti-M2e IgG and IgG isotypes were observed in CC-OMV-M2e-vaccinated mice compared to control mice (Figure 4A). The IgG1:IgG2a ratio was compared between pre- and post-challenge, and an IgG2a bias was observed post-challenge, suggesting a role of a Th1 response in protection against lethal influenza challenge (Figure 4B). Several studies have also shown a significant role of IgA in cross-protection [25,26] and in reducing viral transmission [27,28]. A significant increase in serum IgA titers was observed in CC-OMV-M2e-vaccinated mice compared to control mice post-challenge (Figure 4A). Therefore, the results indicate an increased anti-M2e IgG and IgA response that suggests that an intranasal route of administration of CC-OMV-M2e may have an opportunity to protect against influenza.

### 3.3. Intranasal Vaccination with CC-OMV-M2e Elicits a Th1 Bias Post-Infection and Protects Balb/c Mice against Lethal Influenza A/PR8 Challenge

To explore the type of cell-mediated immune response generated through mucosal immunization, the T cell cytokine response from the spleen was assessed. CD4+ and CD8+ T cells were gated and analyzed for IFN-γ, TNFα, and IL-17 effector cytokine production (Figure S2). Previous reports have shown that effector T cells like Th1 and Th17 are involved in protection against influenza [29,30].

**Figure 5:**
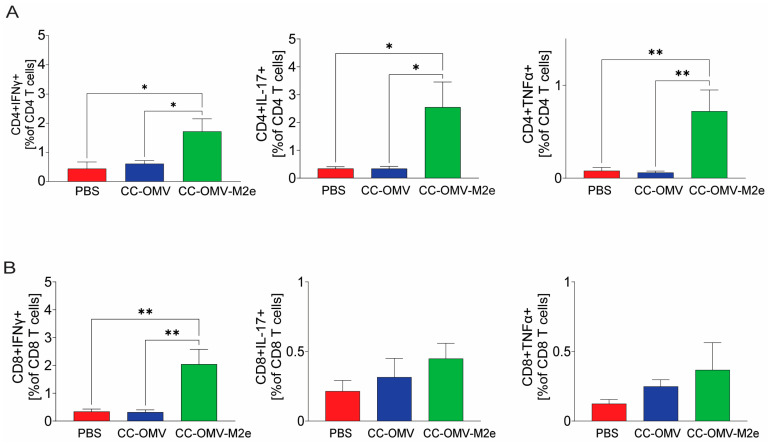
Intracellular cytokine response by CD4+ and CD8+ T cells in the spleen: splenocytes were isolated from mouse spleen on termination. Subsequently, the cells were stimulated for 36 h with 1 µg/mL of CC-OMV-M2e. Analysis of total specific effector T cytokine responses was performed via intracellular flow cytometry. Frequencies of T cells ((**A**) CD4+, (**B**) CD8+) that produce any of the effector cytokines IFN-γ, IL-17, and TNFα upon CC-OMV-M2e stimulation were identified. Results are displayed as % of T cells. Bars represent means ± SEMs with four to five mice in each group (CC-OMV-M2e = 4, CC-OMV *n* = 5, PBS *n* = 5). ** *p* < 0.01 and * *p* < 0.05 as per ordinary one-way ANOVA and Tukey’s post hoc test.

Intranasal immunization induced a significantly higher frequency of IFN-γ producing CD4+ T cells (Figure 5A) and CD8+ T cells (Figure 5B) in the CC-OMV-M2e group relative to the control CC-OMV and PBS groups. Similarly, a significantly higher frequency of TNFα- and IL-17-producing CD4+ T cells was observed in the CC-OMV-M2e group when compared to the control group (Figure 5A). Though an increase in the frequency of TNFα- and IL-17-producing CD8+ T cells was observed in the CC-OMV-M2e group, the trends were not significantly higher than in the controls (Figure 5B). These results indicate that there is a Th1/Th17 bias generated by CC-OMV-M2e via the intranasal mucosal vaccination route.

**Figure 6:**
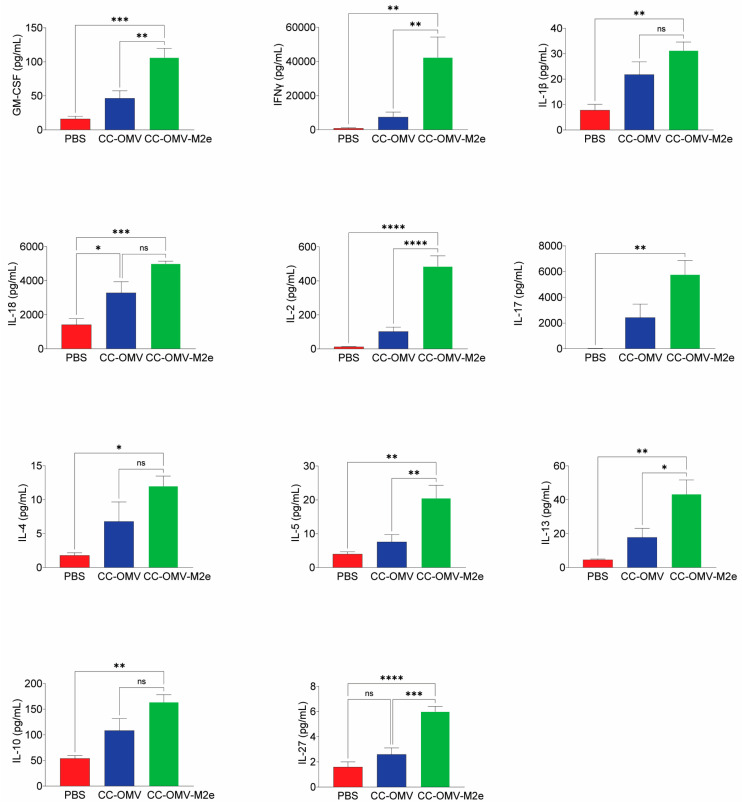
Secreted cytokine response by stimulated splenocytes: splenocytes were isolated from mouse spleen on termination. Subsequently, the cells were stimulated for 36 h with 1 µg/mL of CC-OMV-M2e. Luminex assay was used to quantify the indicated cytokines, a total of 17 of which were tested. Results of 11 out of 17 are displayed as pg/mL. Bars represent means ± SEMs with four to five mice in each group (CC-OMV-M2e group *n* = 4, CC-OMV group *n* = 5, PBS group *n* = 5). * *p* < 0.05, ** *p* < 0.01, *** *p* < 0.001, and **** *p *< 0.0001, ns-non significant, as per ordinary-measures one-way ANOVA and Sidak’s post hoc test.

Several important groups of immune effectors, e.g., cytokines, chemokines, and adhesion molecules, contribute to the production of antibodies and are produced by cells like lymphocytes, macrophages, and dendritic cells. The total cytokine response was assessed from the re-stimulation of splenocytes with CC-OMV-M2e. The concentrations of 17 cytokines (GM-CSF, IFN-γ, IL-1β, IL-2, IL-4, IL-5, IL-6, IL-12p70, IL-13, IL-18, TNFα, IL-9, IL-10, IL-17A, IL-22, IL-23, IL-27) were quantified in the splenocyte culture supernatant using the multiplex bead assay system. The levels of IL-12p70, TNFα, IL-6, IL-9, IL-22, and IL-23 cytokines were not significantly different for CC-OMV-M2e mice relative to the controls. The cytokines IL-1β, IL-4, IL-5, IL-13, and IL-27 were significantly elevated (5–50 pg ⁄ mL) in the supernatant of the CC-OMV-M2e group relative to the controls (Figure 6). Of the 17 cytokines measured, the highest cytokine levels, which were for GM-CSF, IFN-γ, IL-2, IL-18, IL-17A, and IL-10, ranged between 100 and 6000 pg/mL in the supernatant for the CC-OMV-M2e group relative to the controls (Figure 6). IFN-γ, IL-17, and IL-18 had the highest levels of induction amongst all the cytokines tested (above 4000 pg/mL), confirming the likelihood of a Th1/Th17 bias generated in mice vaccinated intranasally with CC-OMV-M2e.

## 4. Discussion

This study was conducted to evaluate and characterize the protective immune response to influenza challenge generated from the intranasal administration of a CC-OMV-M2e formulation. The nasal mucus and cilia present a major challenge in the development of nasal vaccines. They are a natural defense barrier and therefore can obstruct vaccine uptake by antigen-presenting cells. OMVs, owing to their length scale and fluidity, can either be transported paracellularly (between cells) or transcellularly (through cells) through the mucous layer and engage mucosal immune cells.

Lung influenza viral copy numbers were ~2000-fold lower, with significantly higher anti-M2e IgG and IgA antibody titers, in CC-OMV-M2e-vaccinated mice compared to controls. There was a Th1 bias observed based on the IgG1:IgG2a ratio. This result was corroborated by the increased presence of IFN-γ-producing CD4+ and CD8+ T cells in the spleens recovered from CC-OMV-M2e-vaccinated mice compared to controls and the significant production of Th1 cytokines such as IFN-γ, GM-CSF, IL-2, IL-18, and IL-1β in vaccinated mouse splenocytes. In addition, IL-17-producing CD4+ and CD8+ T cells, along with the increased production of the IL-17 cytokine in splenocyte supernatants, were also observed in vaccinated mice.

Traditional parenteral routes of OMV vaccination, which include intramuscular, subcutaneous, and intradermal routes [31], can induce significant systemic protective immune responses. Previous studies employing the CC-OMV-M2e vaccine described herein successfully induced protective immune responses against influenza via the subcutaneous route, a version of which is currently progressing toward Phase I human clinical trials [24]. Although parenteral routes of vaccine administration are effective, the intranasal route is also attractive to generate a greater mucosal immune response and to increase patient acceptance by eliminating the use of needles [20].

Intranasal vaccination is an effective way to elicit an immune response in the respiratory, gastrointestinal, and genital tracts. Like the skin, the mucosa is replete with APCs, which serve to prevent infections through the induction of effective mucosal and systemic immune responses [22]. Upon pathogenic insult, antigens are processed by dendritic cells and other APCs present beneath the epithelium and follicle-associated epithelium, which then migrate to nasopharynx-associated lymphoid tissue (NALT) and interact with CD4+ and CD8+ T cells to induce an immune response [22]. An effective mucosal vaccine will induce the production of neutralizing immunoglobulin A (IgA) and immunoglobulin G (IgG), which, upon subsequent infection, can prevent infection at the site of entry [32]. In addition to the antibody response, vaccines administered via the mucosal route are also able to induce a robust Th1/Th17 response that is protective against influenza infections [33]. Generally, vaccines administered via injection through the skin induce poor IgA responses [34], which reduces their ability to prevent infection at the mucosa.

Influenza-infection-induced Th1 effector cells express antiviral cytokines, such as IFN-γ, TNFα, and IL-2 [35], and activate alveolar macrophages [29]. Th1 cells also regulate the differentiation of CD8+ T cells through IL-2 and IFN-γ secretion to clear viral infection [36,37]. Moreover, it has been demonstrated that Th17 and Tc17 cells mediate protective immunity against highly virulent influenza strains in humans [30]. Observations from Askovich et al. have demonstrated that the early activation of IL-17 production correlates with increased protection against influenza challenge in mice [38].

The M2e antigen is an established epitope that induces protective immunity against lethal challenge with influenza A virus in the mouse model [39]. We observed results similar to those in the literature wherein the CC-OMV-M2e-vaccinated mice survived lethal challenge with influenza A, had commensurate low viral lung titers, and had an enhanced Th1-type M2e-specific IgG antibody response. The IgG subclass distribution (i.e., IgG1 and IgG2a) and the cytokine milieu are indicative of the Th-cell response bias. In the current study, pre-challenged mice had elevated IgG1 titers, but in post-challenge mice, there was an increase in IgG2a titers, indicating a Th1 bias. The literature has established that influenza protection afforded by vaccines containing the M2e antigen is through ADCC and ADCP, leading to the clearance of infected cells, and IgG2a antibodies are involved in the resulting protection in mice [40]. Our results correlate with this mechanism of influenza clearance.

In Balb/c mice, a Th1 response is characterized by a predominantly IgG2a antibody response with elevated IFN-γ and other Th1 cytokines and IFN-γ inducers (GM-CSF, IL-12, TNFα, IL-2, IL-18, IL-1β). CD4+ T cells that are generated in response to viral infection produce a substantial amount of IFN-γ and are designated as a Th1-type phenotype. We observed elevated levels of IFN-γ in the splenocytes of mice vaccinated with the CC-OMV-M2e vaccine. CD4+ T cells not only help promote B-cell antibody production but are also required for the generation of cytotoxic and memory CD8+ T cell populations [41]. We observed a similar response in mice vaccinated with CC-OMV-M2e compared to controls post-challenge. We also observed a similarly high production of IFN-γ from CD8+ T cells, and CD8+ T cells producing IFN-γ are actively involved in viral clearance [41].

Further, there was a significant increase in the levels of IL-17 produced by splenocytes in mice vaccinated with the CC-OMV-M2e vaccine. Previous studies have also shown strong IL-17 responses in mice from both intranasal vaccination [42] and influenza virus challenge [43,44]; however, the exact role of IL-17 in the pathogenesis of influenza and protection is not clear. Recent reports suggest a role for IL-17 in inducing a protective immune response against influenza viral challenge in mice [43,45]. CD4+ IL-17-producing cells, designated as Th17 cells, can enhance the Th1 response and aid in promoting cytotoxic T-cell activity during viral infections [46], and a similar increase in the frequency of IL-17-producing CD4+ T cells was identified in the splenocytes of mice vaccinated with CC-OMV-M2e. Th17 cells can aid in protection through the promotion of enhanced neutrophil infiltration at the site of infection through the upregulation of CXC chemokines that aid in neutrophil recruitment [47] and that contribute to protection against the influenza virus [48].

Increased levels of IL-1β, IL-18, and IL-27 were observed in the splenocytes of mice vaccinated with CC-OMV-M2e. The early induction of Th1 cytokines such as IL-1β and IL-18 is protective to infected hosts by promoting CD8+ T-cell activity and antibody responses [49], and IL-27 is effective against viral infections by decreasing immunopathology and increasing survival [50].

Mucosal IgA antibodies in mice are indicative of cross-clade protection after intranasal vaccine administration [51]. Serum IgA may also be involved in ADCC and ADCP through interaction with the FcαRI receptor [52]. In this study, increased levels of serum IgA were detected in mice post-challenge. Further, the IgA response in vaccinated mice may have been augmented by elevated levels of IL-5 and IL-10, which are cytokines that promote B cells to switch antibody production to IgA and act as anti-inflammatory and regulatory cytokines to control initial inflammation. Elevated levels of IL-4, IL-5, and IL-10 were observed in the splenocytes of mice vaccinated with CC-OMV-M2e. Cytokines such as IL-4, IL-5, IL-6, IL-10, and transforming growth factor-β are critical in the production and maintenance of IgA at the mucosal surface [53,54]. While systemic immune responses to the intranasal vaccine were measured in this study, and provided strong evidence for its protective capability, the mucosal responses of the lung and nasal cavity were not. The mucosal response is a key parameter to fully understanding the mechanism(s) through which the vaccine functions, and reports on these studies are to be provided in a subsequent manuscript.

## 5. Conclusions

Currently, most influenza vaccines are administered intramuscularly and are based on the prevailing seasonal influenza stains. The only available intranasal vaccine is FluMist, a live attenuated vaccine. The protective efficacy of FluMist has been demonstrated to be lower than parenteral administration [55]. To circumvent this problem, we tested a subunit vaccine CC-OMV-M2e through the intranasal route. We showed the increased production of a protective proinflammatory response via the intranasal route through the administration of CC-OMV-M2e, leading to increased survival post-lethal influenza challenge, demonstrating that a potential universal OMV vaccine designed to protect against influenza can be administered through the nasal route.

## Data Availability

Data is contained within the article and Supplementary Material.

## Supplementary Materials

The following supporting information can be downloaded at: https://www.mdpi.com/article/10.3390/vaccines12070724/s1, Figure S1. OMV characterization: (A) M2e antigen content in OMVs was determined via Western blot (WB). Unmodified Western blot shown on the right for full view of the bands on the gel. ClyA-M2e4xHet detected using anti-His antibody (left) and anti-ClyA antibody (right). (B) OMV size characterization: Dynamic light scattering measurements of the average hydrodynamic diameter of OMVs were made using a Malvern Zetasizer Nano ZS. Error bars represent means + SDs. Figure S2. Flow cytometric gating strategy. Single cells were gated on forward scatter-A (FSC-A) vs. FSC-H and side scatter-A(SSC-A) vs. SSC-H, followed by gating on single cells (FSC-A vs. FSC-H, then SSC-A vs. SSC-H). Single cells were then gated for live cells, followed by gating for CD3+ cells. Thereafter, the CD3+ cells were gated for CD4+ and CD8+ T cells, followed by gating for cytokine production (IFN-γ, TNFα, IL-17).
