# Supplementary material for: Intranasal Vaccination with Recombinant TLR2-Active Outer Membrane Vesicles Containing Sequential M2e Epitopes Protects against Lethal Influenza a Challenge
Source: Vaccines (Basel). 2024 Jun 29;12(7):724. doi: 10.3390/vaccines12070724 (PMC11281606; doi:10.3390/vaccines12070724)
Supplement: Supplementary file 1 [file vaccines-12-00724-s001.zip › vaccines-3049112-supplementary.pdf]

vaccines

MDPI

Supplementary Material

Intranasal Vaccination with Recombinant TLR2-Active Outer Membrane Vesicles Containing Sequential M2e Epitopes Protects against Lethal Influenza a Challenge

Nisha Kannan [1], Annette Choi [2], Mariela A. Rivera De Jesus [1], Peter Male Wei [1], Julie Marie Sahler [2], Stephanie Marie Curley [1], Avery August [2], Matthew P. DeLisa [3], Gary R. Whittaker [2] and David Putnam [1,3,*]

[1]Meinig School of Biomedical Engineering, Cornell University, Ithaca, NY 14853, USA
[2]Department of Microbiology and Immunology, Cornell University, Ithaca, NY 14853, USA
[3]Smith School of Chemical and Biomolecular Engineering, Cornell University, Ithaca, NY 14853, USA
* Correspondence: dap43@cornell.edu

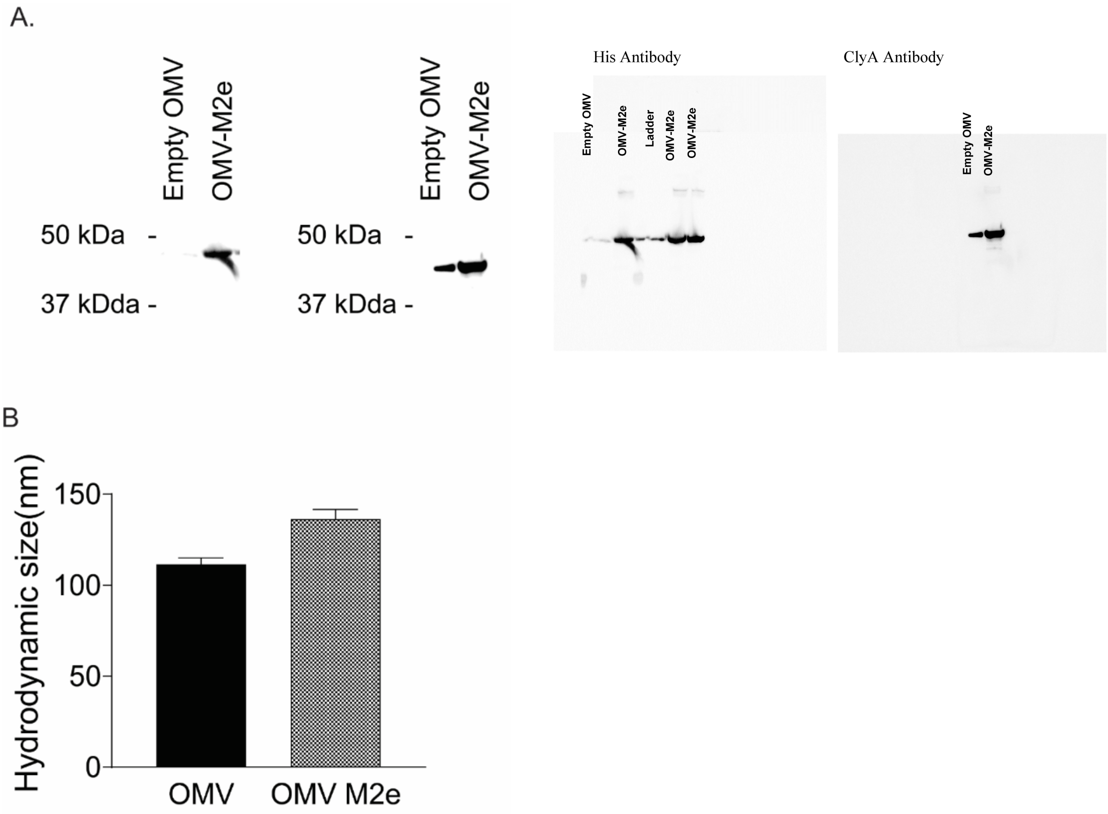

Figure S1. OMV Characterization (A) M2e antigen content in OMVs was determined via Western blot (WB). ClyA-M2e4xHet detected using anti-His antibody (left) and anti-ClyA antibody (right). (B) OMV size characterization: Dynamic light scattering measurements of the average hydrodynamic diameter of OMVs were made using a Malvern Zetasizer Nano ZS. Error bar represent mean± SD.

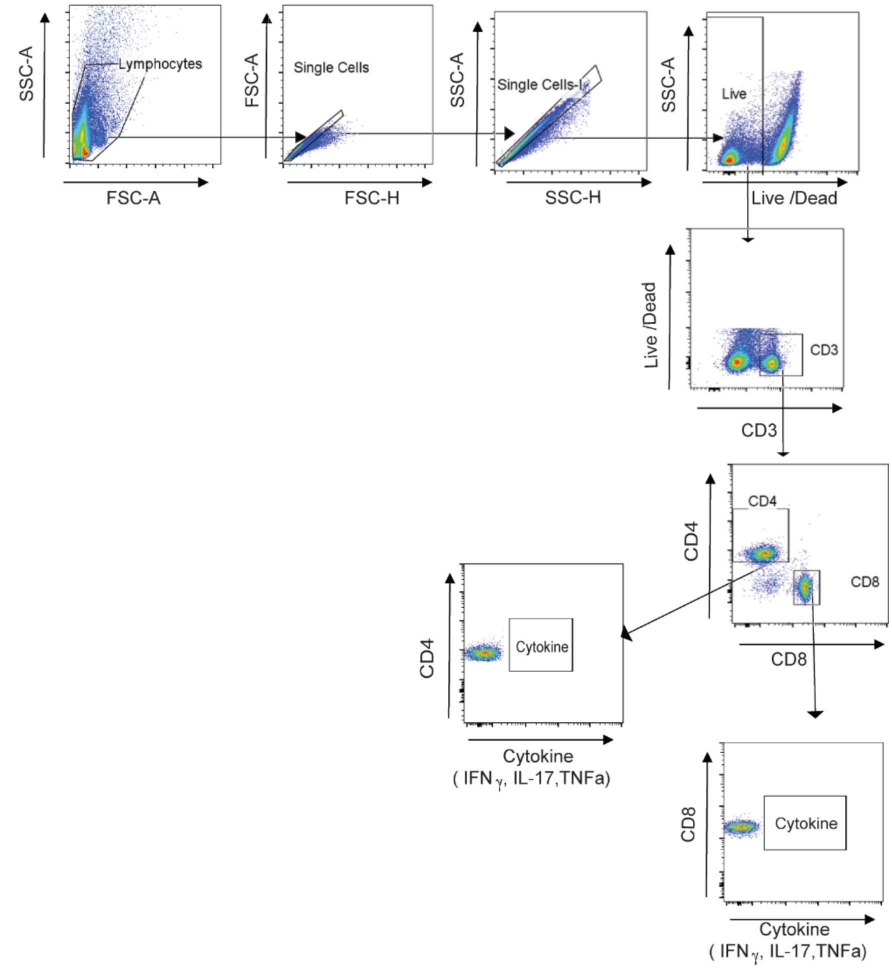

Figure S2. Flow cytometric gating strategy. Single cells were gated on forward scatter- A (FSC-A) vs FSC-H and side scatter -A(SSC-A) vs SSC-H, followed by gating on single cells (FSC-A vs FSC-H then SSC-A vs SSC-H). Single cells were then. gated for live cells, followed by gating for CD3+ cells. Thereafter, the on CD3+ cells were gated for CD4+ and CD8+ T cellss, followed by gating for cytokine production (IFN-γ, TNFα, IL-17).
